# Relationship of ^99m^technetium labelled macroaggregated albumin (^99m^Tc-MAA) uptake by colorectal liver metastases to response following Selective Internal Radiation Therapy (SIRT)

**DOI:** 10.1186/1471-2385-5-7

**Published:** 2005-12-23

**Authors:** Atul Dhabuwala, Prue Lamerton, Richard S Stubbs

**Affiliations:** 1 Wakefield Gastroenterology Centre, Wakefield Hospital, Wellington, New Zealand; 2 Pacific Radiology, Wakefield Hospital, Wellington, New Zealand

## Abstract

**Background:**

SIRT is an emerging treatment for liver tumours which relies on the selective uptake by tumour of ^90^Y microspheres following hepatic arterial injection. Response rates of around 90% are reported. Hepatic arterial injection of MAA gives an indication of the expected distribution of ^90^Y microspheres within the liver. This study sought to determine if the MAA scan could be predictive of subsequent tumour response.

**Methods:**

58 patients with colorectal hepatic metastases received SIRT. All had pre-treatment MAA planar images and CT scans which were retrospectively reviewed. Tumours were qualitatively considered "cold", "equivocal" or "hot" based on MAA uptake and the ratio of uptake in tumour and normal liver tissue was calculated (TNR). Following SIRT (which included the administration of hepatic arterial Angiotensin 2) tumour response was assessed by CEA changes one to two months after treatment and by serial CT.

**Results:**

Uptake was classified as "hot" in 37 patients (Group 1) and "equivocal" or "cold" in 21 (Group 2). CEA levels fell dramatically in over 90% of patients. The falls were not significantly different between the groups. There was no correlation between TNR and tumour response based on CEA changes (r^2 ^= 0.004). CT responses after 3 months were not different in the 2 Groups.

**Conclusion:**

The pattern of MAA uptake by colorectal liver tumours after arterial injection is not a predictor of tumour response after treatment by SIRT. The results suggest the doses of ^90^Y microspheres used may be greater than is necessary.

## Background

Colorectal cancer (CRC) is a particularly common cancer encountered worldwide. Although the primary site is resectable in the majority of instances, metastatic disease will be present or develop in some 50% of those affected within 5 years of presentation. As a result of the portal venous route of spread, the liver is the commonest site of metastases and is frequently the only evident site [1]. This feature of CRC, coupled with the disappointing results achieved with systemic chemotherapy, has lead to interest in the development of effective regional liver therapies. Such regional approaches include liver resection, cryotherapy or radiofrequency ablation, regional chemotherapy and selective internal radiation therapy (SIRT).

SIRT has been shown to be a particularly effective form of local treatment of CRC liver metastases even when large volumes of tumour are present in multiple sites [2,3]. It involves selective delivery to tumours of a high dose of ionising radiation achieved by injecting ^90^Yttrium microspheres directly into the hepatic artery. The microspheres, because of their size (25–35 μm) become trapped in capillary beds and preferential uptake into tumour tissue is achieved because of the predominant arterial blood flow to liver tumours relative to normal liver parenchyma [4]. Radiation dosing is therefore non-homogeneous and the precise dose received will vary within tumour and normal liver tissue and from patient to patient. However average doses received by tumour and normal liver tissue have been calculated to be 200–300Gy and 15–25Gy respectively [5]. A supra-lethal dose of radiation is therefore received by much of and occasionally all of the tumour, while the dose received by the normal liver tissue is insufficient to result in clinically relevant radiation hepatitis. We and others have shown that fewer than 10% of patients with CRC liver metastases will not respond to this form of treatment based on serial CT scanning and tumour marker (CEA) data [2,3]. While definitive evidence of extended survival is not yet available, all data point strongly to some survival advantage, at least for those who do not develop extrahepatic disease at an early stage [2,6]. Selection of those patients most suitable for this form of treatment might be aided by development of a test capable of predicting liver tumour response after SIRT.

^99m^Tc-MAA scans are routinely advised and performed prior to SIRT to confirm access to all areas of the liver from the hepatic artery and isolation of the liver from other foregut structures [7]. ^99m^Tc-MAA has a "particle" size (10–60 μm, average 35 μm) similar to the resin microspheres used for SIRT and can therefore be used as a tracer dose to indicate approximate distribution of ^90^Yttrium microspheres to be expected during SIRT. Tumours with high ^99m^Tc-MAA uptake can be expected to receive a high dose of ^90^Yttrium microspheres and it seems possible they may respond correspondingly better than tumours demonstrating low uptake of ^99m^Tc-MAA. This hypothesis has not previously been thoroughly tested. There have however been contradictory reports regarding the association between ^99m^Tc-MAA uptake by hepatic tumours and response after hepatic artery chemotherapy (HAC) [8-11]. The purpose of this study was to determine whether the pattern of uptake of ^99m^Tc-MAA after arterial injection, by colorectal liver metastases is predictive of tumour response after SIRT.

## Methods

Between February 1997 and February 2000, 61 patients with extensive colorectal liver metastases, not suitable for either resection or cryotherapy, were treated with SIRT. All patients being so treated gave written informed consent for their treatment, as required and approved by the Wellington Regional Ethics Committee. Patients were assessed prior to treatment with CT scans of the chest (or chest Xray), abdomen (and pelvis if appropriate), and standard blood tests including liver function tests (LFTs) and carcinoembryonic antigen (CEA). While CT scans of the chest and bone scans were not routinely performed they were done in many patients. Patients with anything more than minor extrahepatic metastatic disease were excluded from this treatment.

Laparotomy was performed for placement of an hepatic artery Porta-cath^® ^via the gastroduodenal artery in 54 patients, by a previously described technique which entailed ligation of anomalous (duplicated/triplicated) hepatic arteries and small vessels passing to non-hepatic structures [2]. These patients subsequently received a single dose of SIR-spheres^® ^(Sirtex Medical Pty Ltd, Sydney) via this catheter, which was followed by 4-weekly cycles of continuous 5-fluorouracil (5FU) at a dose of 1 g per day for 4 days each cycle. In the remaining 7 patients a hepatic artery catheter (3F Tracker) was placed into the hepatic artery via the femoral artery using a Seldinger technique for the purpose of SIRT. The gastroduodenal artery was embolised unless the catheter could be advanced beyond this artery and still provide access to both left and right hepatic arteries. These patients received a single dose SIR-spheres^® ^via the catheter but did not receive subsequent regional or other chemotherapy. The dose of SIR-spheres^® ^administered was modified according to an estimate of liver tumour volume, based on viewing of the pre-operative CT scan. Thus:

less than 25% liver involvement: 2.0GBq

25–50% liver involvement: 2.5GBq

greater than 50% liver involvement: 3.0GBq

One to two hours prior to SIRT, a ^99m^Tc-MAA liver scan (MAA scan) was undertaken as follows. 100–120 MBq (2.7–3.2mCi) of ^99m^Tc-MAA (Amersham Pulmonate II from UK, particle size 10–60 μm, average 35 μm) in 0.23–0.27 ml was administered into the hepatic artery either via the Port or the percutaneous arterial catheter at a rate of approximately 1 ml/10 sec followed by a flush with 10 ml of normal saline then 5 ml of heparinised saline. Immediately following this injection, images were obtained on a GE Starcam gamma camera. Anterior views of the chest and abdomen and lateral views of the abdomen were acquired for 2 minutes per view on a 256 × 256 matrix with the patient in a supine position. The primary purpose of this scan was to enhance the safety of the subsequent administration of SIR-spheres^®^, by demonstrating the absence of obvious access to extrahepatic sites within the abdomen and by permitting calculation of the degree of liver-lung shunting. It has been shown that if the liver-lung shunt exceeds 13%, there is appreciable risk of radiation pneumonitis and in that event the procedure should be abandoned, or the dose of SIR-spheres^® ^reduced [12].

A few minutes prior to delivery of SIR-spheres^®^, 50 μg Angiotensin II (Hypertensin, Novartis) was injected into the hepatic artery to achieve vasoconstriction of the arterioles to normal liver tissue thereby aiding selective uptake of the microspheres by tumour within the liver. SIRT was administered under light sedation with Hypnovel^® ^(Roche) and intravenous narcotic analgesia by injecting SIR-spheres^®^, into the hepatic artery at a rate of approximately 1 ml/10 sec over some 10 minutes. The technique has been described in more detail elsewhere [2,13].

Blood tests, including albumin, bilirubin, alkaline phosphatase (ALP), alanine transaminase (ALT), and aspartate transaminase (AST) were performed prior to and at 24 and 48 hours following SIRT in the majority of patients. Patients were seen at monthly intervals for review, at which time blood tests including CEA were done. Repeat CT scans were performed at 3-monthly intervals during follow-up. The higher of the values of ALT and AST at 24 or 48 hrs after SIRT was used as an indicator of the degree of injury to normal liver parenchyma. All serum concentrations of CEA and LFTs have been expressed as mean ± sd (confidence interval).

### Assessment of MAA scans

#### (a) Qualitative assessment

This was independently undertaken by two investigators (AD and RSS). Two sketches of the liver were made for each patient by each investigator. On the first was marked the sites of metastatic disease within the liver after viewing the pre-operative CT scan and/or operation note. On the second was marked the sites of most intense uptake of ^99m^Tc-MAA as shown by the MAA scan. The second sketch (MAA uptake) was completed without viewing the first (site of deposits). Each investigator then compared their two sketches for each patient and assessed the liver tumour sites to be "hot", "equivocal" or "cold" as follows:

"hot": greater uptake of ^99m^Tc-MAA by the tumour than the unaffected liver

"equivocal": similar uptake of ^99m^Tc-MAA by the tumour and the unaffected liver

"cold": less uptake of ^99m^Tc-MAA by the tumour than the unaffected liver

In the event of a different assessment being made by the two investigators, as occurred in 13 patients, a joint reassessment was undertaken and a consensus reached. This was not usually difficult. For the purpose of statistical analysis those patients with a "hot" pattern of uptake were termed Group 1 and those with "equivocal" or "cold" uptake were termed Group 2.

#### (b) Quantitative assessment

This was conducted by a third investigator (PL) and entailed a quantitative assessment of the ratio of ^99m^Tc-MAA uptake by tumour and normal liver (TNR). Once again a sketch was made showing the sites of tumour within the liver. The stored computerised images of the MAA scans for each patient were reviewed and a total of 4 to 6 circular regions of interest were drawn on each scan corresponding to sites of tumour and a further 4 to 6 regions of interest corresponding to unaffected normal liver. The diameter of each region of interest was the same for each patient, but varied somewhat between patients (1.0 – 2.6 cm). The TNR for each patient was calculated by dividing the average count for the areas of tumour by the average count for the areas of non-affected liver.

### Assessment of tumour response to SIRT

This was assessed by changes in both CEA and by CT scanning following SIRT. Percentage reduction in CEA was calculated for each patient from the pre-operative level to the lower of the levels at 1 or 2 months following SIRT. This time point was chosen so as to most accurately reflect the response to the SIRT while minimising any effect of ongoing hepatic artery chemotherapy and any effect of subsequent disease progression in the liver or extrahepatic sites. CT changes within the liver were defined as follows:

tumour regression: definite reduction in size of index lesions

stable disease: no definite increase or decrease in lesion size

progressive disease: definite increase in size of index lesions

### Survival

Patient survival was assessed both from time of diagnosis and time of treatment.

### Statistical analysis

Differences in tumour and patient characteristics between groups before and after SIRT were analysed using a paired or unpaired Students t-test or chi-square test as appropriate. Because the CEA data was not normally distributed, all CEA values were log-transformed prior to statistical analysis. The chi-square test was used to assess the significance of differences in response rates judged by serial CT scanning. Estimated survival of patients was calculated by the Kaplan-Meier method and statistical analysis performed using the logrank test. A p value of less than 0.05 was considered significant in all analyses. Linear regression was used to seek any correlation between the tumour response rate (judged by changes in CEA) and TNR uptake of ^99m^Tc-MAA prior to SIRT and the coefficient of determination (r^2^) was calculated.

## Results

Of the 61 patients who received SIRT for colorectal liver metastases, three patients were excluded from this study because pre-treatment CT scans were not available for comparison with the pre-SIRT MAA scan. Thus the study group included 58 patients (38 males and 20 females), with a median age at treatment of 61 years (range 33–76). The median time from diagnosis of colorectal liver metastases to treatment with SIRT was 2.8 months (range 0.5 – 71.6).

### ^99m^Tc-MAA uptake by CRC hepatic metastases

**Figure 1:**
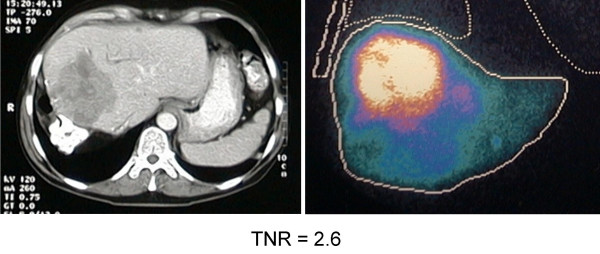
Example showing scans of a patient with MAA uptake described as "hot".

**Figure 2:**
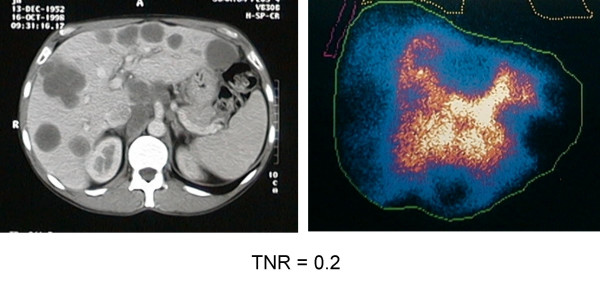
Example showing scans of a patient with MAA uptake described as "equivocal".

**Figure 3:**
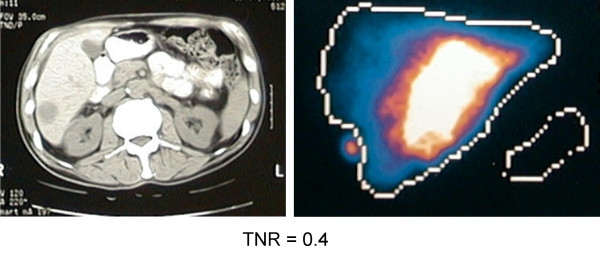
Example showing scans of a patient with MAA uptake described as "cold".

The pattern of uptake of ^99m^Tc-MAA by the liver was "hot" in 37 patients (Group 1). In the remaining 21 patients (Group 2), the pattern was "equivocal" in 12, and "cold" in 9 patients. Examples of these various patterns of uptake are given in Figures 1, 2, 3.

Patient demographics, tumour characteristics and details concerning treatment given are shown in Table 1. There was no significant difference in any of these features between patients from Groups 1 and 2. Pre-treatment liver function tests are shown in Table 2. There was no significant difference in the levels of bilirubin, ALP, ALT, or AST between the two groups, however albumin levels were significantly lower in Group 1 patients than those of Group 2 (p = 0.01). Changes in ALT and AST levels in the 48 hour period following SIRT are shown in Table 3. This data was not available for 8 patients. A significant rise in AST but not ALT was noted in patients from both Group 1 (p = 0.03) and Group 2 (p = 0.05) following SIRT. There was however no significant difference between the rises of either AST or ALT between the two groups.

**Table 1:** Patient demographics and tumour pathology.

	**Group 1**	**Group 2**	
	hot (n = 37)	equivocal (n = 12)	cold (n = 9)

Age years median (range)	61 (33–76)	56 (40 – 72)	64 (50 – 75)
Gender			
Female	21	1	3
Male	16	11	6
ACPS* staging of colonic primary			
A3	1	0	0
B1 & B2	6	1	4
C1 & C2	29	10	4
Unknown	1	1	1
Histology of colonic primary			
well differentiated	1	2	0
moderately differentiated	28	4	6
poorly differentiated	7	2	2
unknown	1	4	1
Liver tumour diagnosis			
synchronous	27	6	5
metachronous	10	6	4
Estimated liver involvement			
<25%	19	5	6
25–50%	8	5	1
>50%	10	2	2
Dose of ^90^Yttrium given			
2.0 GBq	21	6	7
2.5 GBq	10	4	2
3.0 GBq	6	2	0
Liver / lung shunt, median (range)	0.3% (0 – 9.3%)	0.2% (<0.1 – 3%)	0.2% (0.1 – 1.5%)
HAC given after SIRT	34	10	8

* Australian clinico-pathogical staging

**Table 2:** Serum liver function tests before SIRT [mean ± sd (confidence interval)].

	**Group 1**	**Group 2**	
	"hot"	"equivocal"	"cold"

number of patients	37	12	9
albumin g/L	36 ± 6 (34 – 38)*	38 ± 4 (34 – 41)	41 ± 4 (38 – 44)
bilirubin umol/L	16 ± 12 (12 – 20)	16 ± 17 (6 – 26)	11 ± 7 (6 – 16)
ALP iu/L	309 ± 335 (201 – 417)	272 ± 149 (188 – 356)	194 ± 139 (-12357 – 12745)
ALT iu/L	92 ± 122 (53 – 131)	60 ± 53 (30 – 90)	51 ± 35 (28 – 74)
AST iu/L	83 ± 149 (35 – 131)	59 ± 44 (34 – 84)	43 ± 34 (21 – 65)

* p = 0.01 between Groups 1 and 2

**Table 3:** Serum transaminase levels before and after SIRT (paired data) [mean ± sd (confidence interval)]. AST levels rose significantly after SIRT in both Groups but there was no significant rise in ALT following SIRT in either Group. There was no significant difference in the rise of ALT or AST after SIRT between the two Groups.

	**Group 1**	**Group 2**	
	"hot"	"equivocal"	"cold"

number of patients	31/37	12/12	7/9
ALT iu/L			
pre-SIRT	97 ± 128 (52 – 142)	60 ± 53 (30 – 90)	56 ± 36 (30 – 83)
post-SIRT	146 ± 121 (103 – 188)	263 ± 316 (84 – 442)	1665 ± 2517 (-199 – 3530)
			
AST iu/L			
pre-SIRT	88 ± 162 (31 – 145)	59 ± 44 (34 – 84)	48 ± 37 (20 – 75)
post-SIRT	200 ± 205 (128 – 272)	379 ± 437 (131 – 626)	1657 ± 2562 (-241 – 3555)

### Relationship between ^99m^Tc-MAA uptake and tumour response

#### Tumour response assessed by CEA changes

Four patients did not have CEA levels measured after SIRT because of their deteriorating condition. Details of CEA levels in Group 1 and 2 patients are shown in Table 4. Thirty four patients from Group 1 had elevated CEA levels prior to SIRT and 3 patients had normal levels. Following SIRT, 29 of these showed a fall in CEA by 2 months, 3 showed a rise, 2 continued to maintain normal levels and in 3 the CEA changes were not assessed. Twenty patients from Group 2 had elevated CEA levels prior to SIRT and 1 patient had a normal CEA level. Following SIRT, 18 of these showed a fall in CEA by 2 months, 1 showed a rise, 1 continued to maintain normal levels and in 1 the CEA level was not assessed. A significant fall in CEA was observed in both Group 1 and 2 patients over the 2 months following SIRT (p <0.001) indicating tumour responses in both groups. There was no significant difference between the pre-SIRT CEA level (p = 0.06) or the lowest CEA level 1 or 2 months after SIRT (p = 0.06), between the two groups.

**Table 4:** Serum CEA levels before SIRT and the lowest level either 1 or 2 months after SIRT. A significant fall in CEA was seen in both groups after SIRT. The difference between the fall after SIRT in the two groups was not statistically significant.

	**Group 1**	**Group 2**	
	"hot"	"equivocal"	"cold"

CEA (ng/ml) pre-SIRT			
number of patients	37	12	9
mean ± sd (C.I.)	2071 ± 5598 (267 – 3874)	433 ± 628 (77 – 789)	247 ± 390 (-8 – 502)
median (range)	235 (1.1 – 25620)	113 (1.8 – 1749)	41 (4.3 – 1150)
			
CEA (ng/ml) post-SIRT			
number of patients	34/37	12/12	8/9
mean ± sd (C.I.)	428 ± 983 (97 – 758)	50 ± 55 (19 – 81)	41 ± 75 (-14 – 97)
median (range)	15 (1.2 – 4590)	42 (1.9 – 170)	6 (0.8 – 216)

#### Tumour response assessed by CT changes

Seven patients did not have a follow-up CT scan after SIRT because of their deteriorating condition. Tumour responses judged by CT scan 3 months after SIRT are shown in Table 5. Some tumour regression was seen in the majority of patients from both groups with tumour progression being seen in only 3/51 (6%) patients. There was no significant difference between the proportion of CT responses observed in the 2 groups.

**Table 5:** Tumour response assessed by CT changes 3 months after SIRT. There was no significant difference between the proportion of responses seen in the two groups.

	**Group 1**	**Group 2**	
	"hot"	"equivocal"	"cold"

number of patients	33	10	8
tumour regression	24	8	6
stable disease	7	2	1
progressive disease	2	0	1

**Figure 4:**
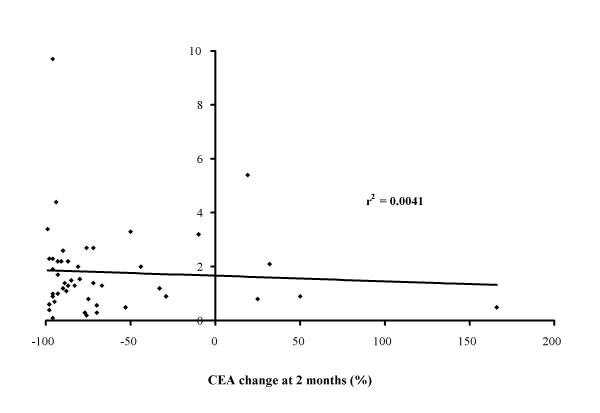
Linear regression line showing no correlation between TNR uptake of MAA and tumour response as assessed by the CEA change after one or two months (whichever is the greater) after SIRT.

Quantitative assessment of TNR showed a mean of 0.5 for the "cold" lesions, 1.0 for the "equivocal" lesions and 2.4 for the "hot" lesions. The mean ± sd TNR for all patients was 1.83 ± 1.56. For Group 1 it was 2.4 ± 1.6 and for Group 2 it was 0.8 ± 0.5. These differences were statistically significant (p = 0.0002). There was however no significant correlation between the TNR and the tumour response as judged by the percentage change in CEA 1 to 2 months after SIRT (see Figure 4). The coefficient of determination for this relationship was 0.004.

### Survival after liver tumour diagnosis and SIRT

Median follow-up for all patients after SIRT is 11.6 months (range 1 – 50.2). Median survival from diagnosis of liver metastases for all patients is 14.1 months (range 1.9 – 91.4) and from treatment is 11 months (range 1.0 – 50.2). Kaplan-Meier cumulative proportion surviving ± se after SIRT is 75.8% ± 6 at 6 months, 50.0% ± 7 at 12 months, 34.5% ± 6 at 18 months, 20.1% ± 6 at 24 months, and 12.1% ± 4 at 30 months.

**Figure 5:**
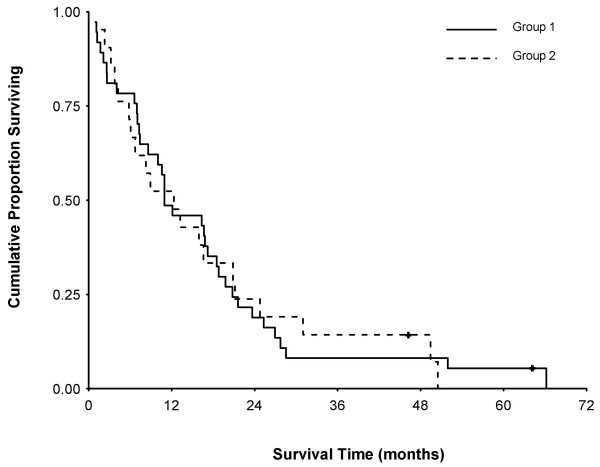
Kaplan-Meier survival curves for Group 1 patients ("hot" lesions) and Group 2 patients ("equivocal" or "cold" lesions). There is no statistical difference in cumulative survival by logrank test.

A comparison of the estimated survival following treatment for Groups 1 and 2 patients is shown in Figure 5. For Group 1 the median survival from diagnosis of liver metastases is 16 months (range 1.9 – 91.4). Median survival time from treatment is 11 months (range 1.0 – 48.5) and the cumulative proportion surviving ± se at 6 months is 78.4% ± 7, 48.6% ± 8 at 12 months, 35.1% ± 8 at 18 months, and 18.9 % ± 6 at 24 months. For Group 2 the median survival from diagnosis of liver metastases is 14.1 months (range 5.4 – 75.8). Median survival time from treatment is 12.3 months (1.9 – 50.2) and the cumulative proportion surviving ± se at 6 months is 71.4% ± 10, 50.0% ± 11 at 12 months, 31.8% ± 10 at 18 months, and 22.7% ± 9 at 24 months. The difference in survival for patients in Group 1 and Group 2 is not statistically significant.

## Discussion

Selective Internal Radiation Therapy is an emerging regional approach to the treatment of liver cancer. Unlike other regional ablative techniques such as cryotherapy, radiofrequency ablation, and laser ablation SIRT is appropriate and efficacious for both small volume and large volume non-resectable disease. Reported response rates are around 90% and in most instances substantial tumour destruction is achieved. Tumour marker data suggests a median destruction of 85–90% of liver tumour by a single treatment with SIRT [2,3]. The cancers with which most experience has been obtained to date are colorectal liver metastases and primary hepatocellular cancer. In the case of the former, the only other local treatment suitable for patients irrespective of the extent of liver involvement is regional chemotherapy. While there is considerable data supporting the use of regional chemotherapy, results are not as reliable or as impressive as with SIRT. The one randomised trial which has been performed comparing regional chemotherapy with SIRT plus regional chemotherapy in patients with advanced CRC liver metastases showed benefit from the addition of SIRT both in terms of response rates and survival [6].

To date experience with SIRT has been confined to a relatively small number of centres with published reports of treatment in over 20 patients coming from very few groups [2,3,5,14-16]. Experience however, is accumulating and has been reported with two types of ^90^Yttrium microspheres – a resin based microsphere (SIR-spheres^®^) [2,3,5] and a glass microsphere (Therasphere^®^) [14-16]. While excellent response rates have been reported for both, more detailed information regarding treatment and outcomes with resin microspheres is currently available. However, much more work needs to be done to refine selection of patients, and treatment schedules.

MAA scans are currently performed as a routine prior to SIRT for the purpose of confirming access to the liver by the delivery system, excluding access to extrahepatic foregut sites, and defining the extent of any liver/lung shunt. This is important for minimising the risk of potentially fatal adverse effects such as radiation pneumonitis or radiation gastritis/duodenitis [12,17]. As MAA particles are of similar size to SIR-spheres^® ^it is reasonable to assume the two will be distributed similarly within the liver. The present study set out to determine whether the pattern or degree of MAA uptake by liver tumours after hepatic arterial injection might be a predictor of response to subsequent SIRT with SIR-spheres^®^. It seemed likely the results of such a study would have potentially important implications regarding selection of patients for SIRT or the determination of an appropriate dosing schedule. At the time this study was undertaken SPECT imaging was not available to us, as indeed it is still not for many of the centres undertaking SIRT. The use of simple planar images for the quantitative element of this study brings a degree of approximation to the measurement of the MAA uptake, which must be acknowledged, but which the authors believe does not alter the underlying message of the study. This belief is based on knowledge and experience gained by the authors over the last two years during which time SPECT/CT imaging of MAA studies has been available to us.

There is an important difference in the blood supply of liver tumours and normal liver parenchyma which is exploited by regional hepatic arterial delivery of anticancer agents such as chemotherapy or ^90^Yttrium microspheres. Malignant liver tumours derive some 95% of their blood supply from the hepatic artery, whereas only 25–30% of the blood supply of normal parenchyma is from the hepatic artery [4]. The balance comes from the portal vein. MAA perfusion scans should give a reasonably reliable indication of the relative arterial flow to liver tumour and normal liver parenchyma. Theoretically, a higher degree of vascularity within the tumour will result in greater arterial blood flow (relative to normal liver parenchyma) and therefore greater receipt of any therapeutic agent delivered via the hepatic artery. The present study reveals considerable variation in the arterial blood flow to CRC liver metastases relative to normal liver with TNR ranging from a low of 0.1 to a high of 9.7 with a mean of 1.83. The majority (37/58 or 64%) of CRC liver metastases do have greater arterial blood flow than normal liver and therefore appear "hot" on an MAA perfusion scan. Patient characteristics and tumour stage, grade and extent do not appear to influence tumour vascularity as judged by TNR.

Early studies of internal radiation therapy reported that patients with relatively avascular tumours, as demonstrated by arteriography, did not respond as well as those patients with moderately to highly vascular tumours [18,19]. These studies, however, involved relatively small numbers and included patients with a variety of different primary cancers. Furthermore arteriography may not be a particularly accurate way of assessing tumour blood flow. The relationship between vascularity of CRC hepatic tumours and response to HAC has been studied by a number of groups. An early study by Kim et al observed that the more vascular the hepatic tumour on angiogram, the better the prognosis following hepatic artery ligation and infusional chemotherapy [20]. Subsequently, a number of authors [8-10] described a correlation between perfusion of CRC liver metastases, measured by ^99m^Tc-MAA scans, and response to HAC. They concluded that increased tumour perfusion allows for a higher response rate from this particular treatment modality. Lehner et al on the other hand, found no correlation between tumour vascularity, as indicated by ^99m^Tc-MAA scan, and response or survival following HAC in a study involving 36 patients with CRC liver metastases. They suggested that the degree of neo-vascularisation may only be demonstrated by super-selective catheterisation and questioned whether perfusion studies by angiogram or ^99m^Tc-MAA reliably reproduce the perfusion pattern produced by the much slower flow rate used for chemotherapy [11]. In a relatively recent publication Dancey et al reported a better response of non-resectable HCC to SIRT with Theraspheres^® ^for patients with "hot" lesions on MAA scan, than those with "cold" lesions [15].

The present study relates to SIRT in CRC metastases and has adopted a more methodical approach to assessing the question than that taken in Dancey's report of SIRT in HCC, and includes a larger number of patients. Not only have we attempted to quantify the MAA uptake more precisely, but we have also attempted to quantify the tumour response more precisely using changes in tumour marker after SIRT. The findings are clear. With the doses used there is no correlation between MAA uptake into CRC liver metastases prior to SIRT (TNR) and response to the treatment in terms of tumour marker data, CT data or survival time. Intuitively, one might have expected the opposite finding. The most obvious implication of this is that the doses of SIR-spheres^® ^being administered may be higher than is required for obtaining a response in most patients. This possibility should be investigated. Comparison between the findings of our and Dancey's study suggests a higher dose of ^90^Y microspheres is required for therapeutic effect in HCC than CRC. The fact that the SIR-spheres^® ^were administered to the patients after giving angiotensin 2 into the hepatic artery, whereas the MAA perfusion scans were performed without angiotensin 2, potentially confounds the results and is unfortunately a weakness arising out of the retrospective nature of the study. The vasoconstriction achieved by angiotensin 2 has been shown to assist selective tumour uptake [21] and provides the theoretical basis for its use. Most units delivering SIRT do not use angiotensin 2 as it is no longer readily available and this does not obviously compromise results. The authors feel it is unlikely that the lack of correlation between TNR uptake of MAA and tumour response to SIRT would be accounted for by the administration of angiotensin 2, but it remains a possibility. If a positive correlation between the pattern of MAA uptake and tumour response was subsequently shown by another group, not using angiotensin 2, an argument might be raised supporting the routine use of a vasoconstrictor during SIRT. We currently use a bolus dose of phenylephrine hydrochloride 100 μg in lieu of angiotensin 2, on the grounds this may be of value, and no harm is conferred.

We have observed and previously reported that patients receiving SIRT using the dosing schedule of the present study suffer profound lethargy and anorexia for up to 4–6 weeks after the treatment [2]. The present study provides data to indicate that damage is sustained by normal hepatocytes, as indicated by a significant transaminase rise, after SIRT. There is however, no indication that the extent of this damage relates to the TNR of MAA uptake. Again this suggests that the doses being given are sufficiently great to result in liver damage occurring in a non dose-dependent fashion. If the adverse effects of anorexia and lethargy relate to the damage of normal liver tissue, as seems possible, then a smaller dose of SIR-spheres^® ^might be better tolerated without compromising anti-tumour-efficacy. It follows that a dosing schedule employing repeated smaller doses of SIR-spheres^® ^might be better tolerated than present schedules while achieving maintenance of a high response rate. Again this possibility is worthy of investigation.

## Conclusion

The present study confirms the high rate of response of CRC liver metastases to SIRT reported previously by our group and others. However, it finds no relationship between the pattern of MAA uptake by metastatic colorectal liver tumours and response obtained, at least for the doses of SIR-spheres^® ^administrated in this study.

## Competing interests

RSS has for a number of years been sponsored by Sirtex Pty Ltd at a variety of international and national meetings, for which expenses have been payed by that company. In addition he has been a regular speaker for Sirtex Pty Ltd at company organised/sponsored meetings for which he has received honoraria.

## Authors' contributions

AD participated in the design of the study and the qualitative scan assessments, and helped draft the manuscript. PL Performed the quantitative scan assessments and helped draft the manuscript. RSS conceived of the study, participated in the study design and coordination, participated in the qualitative scan assessments and helped draft the manuscript. All authors read and approved the final manuscript.

## Pre-publication history

The pre-publication history for this paper can be accessed here:


